# An investigation to assess ankle mobility in healthy individuals from the application of multi-component compression bandages and compression hosiery

**DOI:** 10.1186/s13047-016-0151-8

**Published:** 2016-07-07

**Authors:** Leanne Atkin, John Stephenson, Grace Parfitt, Sarah Reel, Karen Ousey, Brandon Fallon

**Affiliations:** School of Human and Health Sciences, University of Huddersfield, Huddersfield, UK; Vascular Nurse Specialist, Mid Yorkshire NHS Trust, Huddersfield, UK; Biomedical Statistics, School of Human and Health Sciences, University of Huddersfield, Huddersfield, UK; Podiatry, School of Human and Health Sciences, University of Huddersfield, Huddersfield, UK; Advancing Clinical Practice, School of Human and Health Sciences, University of Huddersfield, Huddersfield, UK

**Keywords:** Range of ankle motion, Plantarflexion, Dorsiflexion, Venous ulceration, Compression therapy

## Abstract

**Background:**

An investigation was undertaken to compare the effect of multi-component compression bandages and compression hosiery kits on individuals’ range of ankle motion whilst wearing typical and medical footwear, and barefoot.

**Methods:**

A convenience sample of 30 healthy individuals recruited from the staff and student population at the University of Huddersfield, UK. Plantarflexion/dorsiflexion range of ankle motion (ROAM) was measured in participants over 6 steps in every combination of typical, medical and no footwear; and multi-component bandages, compression hosiery and no garments.

**Results:**

Controlling for age, gender and garments, the use of typical footwear was associated with a mean increase in ROAM of 2.54° at best estimate compared with barefoot; the use of medical footwear was associated with a mean decrease in ROAM of 1.12° at best estimate compared with barefoot. Controlling for age, gender and footwear, the use of bandaging was associated with a mean decrease in ROAM of 2.51° at best estimate compared with no garments. Controlling for age, gender and footwear, the use of hosiery was not associated with a significant change in ROAM compared with no garments.

**Conclusions:**

Bandages appear to restrict ROAM more than hosiery when used in conjunction with a variety of footwear types.

**Electronic supplementary material:**

The online version of this article (doi:10.1186/s13047-016-0151-8) contains supplementary material, which is available to authorized users.

## Background

For many years compression therapy has been recognised as the gold standard treatment for venous leg ulceration [1]. Compression therapy can be delivered in a variety of forms; the most commonly used are multi-component compression bandages and compression hosiery kits. Compression hosiery kits consist of two layers of stocking, which, when applied, have a low profile which does not restrict patients’ choice of footwear or clothing; whereas multi-component compression bandages normally consist of four separate bandages, and the bulk associated with these can restrict footwear and clothing choices. Both have been proven to aid healing of venous ulceration [2]. In terms of healing rates, there is no significant difference between the two treatment options [2]. However, there are some reported advantages and disadvantages of the two systems: compared to hosiery kits, bandages tend to be bulkier and more expensive over the duration of treatment, but can be tolerated by more patients as some participants in research trials found hosiery kits uncomfortable [2].

Any restriction on ankle movement will affect balance and gait, and this could introduce a risk of falling [3]. A sample of 176 elderly volunteers (mean age 80.1 years) was analysed [4] to identify foot and ankle features that could contribute to falls; finding that ROAM was a significant independent predictor of falls in the elderly (*p* < 0.01). In a follow-up study [5], the same cohort of volunteers were assessed for incidence of falling and placed into 2 groups – those who fell to the ground on at least one occasion during a twelve month follow-up period (*n* = 71) and those who did not fall (*n* = 104). There was a significant difference between fallers and non-fallers in terms of ankle flexibility, in agreement with other prospective studies [3, 6–8].

National Institute Clinical Excellence (NICE) guidelines have identified that ROAM can be modified; thereby reducing the risk of falls using strength and balance training, such as participating in Tai Chi, group or individual foot and ankle strengthening programmes [9, 10]. However, if ROAM is impeded by compression bandaging, such modifications to reduce the risk of falling may not produce expected improvements as ROAM will remain limited in these patients. By contrast, hosiery kits may not be as restrictive.

A recent literature review [11] states that little is known about the risk of falling for people with venous leg ulceration. It has been postulated [11] that patients with venous ulceration are at significantly higher risk of falling owing to reduced balance and mobility.

Multi-component bandage systems worn by patients with venous leg ulceration can cause footwear-related problems. Often patients’ normal footwear will not accommodate the compression bandages, which may require medical footwear. An audit of community patients prescribed compression bandages found that 26 % of patients wore only socks, slippers or even went barefoot on account of the bulk of the compression bandages, with a further 32 % being able to use only open-toe shoes or sandals as footwear [12]. Footwear alone has been identified as an environmental risk factor for falls; the use of sub-optimal footwear, and walking indoors barefoot or in socks has been shown to increase the risk of falls in older people [13]. Compression hosiery kits do not restrict patients from using their normal footwear, due to the low profile, lightness and lack of bulk; and so can eliminate some of these factors which result in increased fall risk.

Hence the potential footwear implications and changes in ROAM arising from the use of multi-component compression bandages may affect patients’ risk of falling. This study therefore compares the effect on ROAM of multi-component compression bandages and compression hosiery kits, measured over different types of footwear, focussing on the sagittal plane movements’ dorsiflexion and plantarflexion of the ankle (talo-crural) joint.

## Methods

### Participants

Participants were recruited from the staff and students at the University of Huddersfield using convenience sampling. Participants were excluded from the study if they had had a lower limb injury in the previous 5 years.

### Data collection

Testing was conducted in the biomechanics laboratory at the University of Huddersfield, School of Human and Health Sciences. The primary outcome measure for each participant assessment was ankle joint dorsiflexion and plantarflexion range of movement during walking. This was captured using SimiMotion Version 9.0.3, 2-dimentional (2-D) analysis.

Participants were assessed in the conditions of barefoot, wearing typical footwear and wearing medical footwear. Within each of these conditions, participants were assessed with a compression hosiery, with multi-component compression bandaging, and with no compression hosiery or garments. Hence each combination of footwear and hosiery was represented; amounting to 9 assessments per participant.

BSN medical UK provided the JOBST Comprifore multi-layer bandage kits and JOBST UlcerCARE compression hosiery kits in the required sizes for all participants. Both vascular treatments provide sustained and graduated compression of 40 mmHg. All the bandages were applied by the same qualified nurse. Participants’ calf and ankle circumferences were measured to establish their suitable compression hosiery size following the guidelines. The medical footwear used for the study was a BeneFoot “Original” post-operative shoe, which is representative of what is supplied in clinical practice.

Participants were assessed walking on an uninclined Bremshey RN5 treadmill; allowing a sufficient amount of steps in a normal walking pattern to be adopted by participants without having to change direction.

The study used a high speed video camera from Matrix vision, model mvBlueCOUGAR (2048x1048 pixels). The camera was elevated on a tripod to the height of 25.5 cm and was positioned in the centre adjacent to the treadmill at a distance of 104 cm to record the participants’ right lower limb in the sagittal plane. Calibration was captured for 2-D space by a known calibration object measuring 0.38 m in height and 0.48 m in width to ensure the accuracy of the camera set up for data collection. The system allowed capture of information at a rate of 100 Hz.

A qualified podiatrist applied reflective skin markers to each participant to allow the calculation of the participants’ talo-crural joint kinematics. The locations of the markers included the bony prominences of the lateral epicondyle (femur), lateral malleoli, peroneal trochlea, styloid process, and the head of the 5^th^ metatarsal. The locations of these markers are consistent with other 2-D studies measuring ankle range of movement and the sagittal points in 3-D studies [14–17]. These coordinates correspond to those used in routine clinical practice when measuring ankle range of movement during visual estimation and with the use of measuring devices such as goniometers [16, 18].

The assessment began when each participant had settled into their own stable walking pattern and pace. This time allowance included at least 5 steps before recording commenced. This is representative of the mid-gait protocol which is considered the gold standard in pressure analysis data collection [19]. Beginning the assessment after 4 or more steps improves the reliability of capturing the participants’ natural walking style rather than recording imminently [20, 21]. Up to 6 steps were captured for each condition. For each step ROAM was calculated by subtraction of the minimum recorded angle from the maximum recorded angle. To standardize the procedure, only the participants’ lateral aspect of the right ankle joint movement was assessed in all of the conditions. Participants’ dominant leg was recorded to assess whether this had an influence, along with other demographic information such as height and weight (from which body mass index (BMI) was calculated), age and gender.

The reflective markers were automatically tracked and visually checked by a technician to ensure no errors were made. Dorsiflexion and plantarflexion movement data was then exported into Microsoft Excel to calculate participants’ range of movement in each of the 9 conditions Additional file 1.

### Statistical analysis

Following data cleaning processes, the sample was summarised descriptively. A series of random intercepts multilevel regression analyses (with parameters estimated by the iterative generalised least squares method) were conducted to assess the effect of footwear and garments; and the controlling variables of age, gender and BMI on the ROAM outcome measure. Participants were considered to form the upper level of the analysis, assessments nested within participants were considered to form the middle level of the analysis and steps nested within assessments were considered to form the lower level of the analysis. Demographic variables were defined at “participant” level; footwear and garment variables were defined at “assessment” level. Reference categories were defined for the factors of footwear and garments to be the “barefoot” state and the “no garments” state respectively.

Initially the controlling variables were considered on an individual basis in one-at-a-time analyses: any variable whose omission resulted in a substantive reduction in goodness-of-fit was carried forward for inclusion in multiple analyses also including indicator variables corresponding to typical and medical footwear; multi-component bandages and compression hosiery; and the first-order interactions between each footwear variable and each garment variable.

Interactions were tested on a one-at-a-time basis in the presence of all main effects: any interaction whose omission resulted in a significant and substantive reduction in goodness-of-fit was carried forward for inclusion in a multiple analysis which included all such interactions. Any interactions not associated with a significant or substantive improvement in goodness-of-fit in the presence of other interactions were deleted from the final model. Goodness-of-fit in all models was measured by changes in the likelihood ratio statistic (LRS) between 2 nested models with ν_1_ and ν_2_ degrees of freedom, which approximately follows a *χ*^2^ distribution on ν_1_ - ν_2_ degrees of freedom.

*P*-values and parameter estimates with associated 95 % confidence intervals and effect sizes were reported for all measured factors. The variance partition component for the final model was also calculated to assess the relative components of variation: between participants; between assessments within participants; and between steps within assessments. All data was analysed using SPSS statistical software (version 22).

## Results

Data was obtained from 30 healthy participants (21 females, 9 males), with mean age of 35.0 years (SD 10.7 years; range 20–59 years), and mean BMI of 26.5 kg/m^2^ (SD 5.46 kg/m^2^; range 18.4–38.0 kg/m^2^). Twenty four participants reported their right leg to be their dominant leg; 5 participants reported their left leg to be their dominant leg.

Fourteen participants could not be assessed wearing both compression bandages and typical footwear, as the bandaging was too bulky to allow the shoe to fit. The wearing of hosiery did not impede any participants from wearing footwear of any kind. Data was not obtained from one participant in the barefoot state due to equipment failure. A small additional number of data items were recorded as missing. A total of 1516 values were reported; missing data values were not imputed. A small amount of obvious transcription and mis-recording errors were corrected as part of the data cleaning process.

The mean recorded ROAM was 24.5° (SD 6.65°; range 0.19° to 50.8°). The absolute maximum recorded angle was 157.6°. The absolute minimum recorded angle (observed in a different participant) was 85.9°.

Screening multilevel models including only the controlling variables of age, gender and BMI on a one-at-a-time basis revealed that inclusion of either age or gender substantively improved model fit according to changes in the likelihood ratio statistic (ΔLRS) compared with a null model. Hence these variables were carried forward for inclusion in subsequent models. The omission of BMI did not result in a substantive ΔLRS compared with a null model; hence this variable was not carried forward for inclusion in subsequent models. Dominant leg was not included in any analysis, due to the paucity of participants with left leg dominance, and the fact that participants were not specifically instructed to begin each assessment on either dominant or non-dominant leg.

Testing of interactions on a one-at-a-time basis in the presence of main effects revealed none of the first-order footwear × garments interactions were associated with significant improvements in model goodness-of-fit. Hence the final model the main effects indicator variables for garments and footwear, plus the controlling variables of age and gender.

Partitioning of variance in the final model revealed that 46.6 % of residual variance was found at the participant level; 29.5 % at the assessment level; and 23.9 % at the step level. Hence nearly half the total variance was accounted for by differences in gait patterns between participants; and nearly a quarter of total variance was accounted for by differences between steps taken by participants in a particular assessment. The remainder, about 30 % of the total variance, was accounted for differences in assessments; i.e. variation in the types of footwear and garments worn.

Model parameters are summarised in Table 1. Controlling for other variables, compared to the state of no garments, bandaging significantly reduced ROAM; while hosiery did not significantly affect ROAM. Controlling for other variables, both typical footwear and medical footwear significantly affected ROAM compared to the barefoot state; with ROAM increased by the use of typical footwear and decreased by the use of medical footwear. Controlling for other variables, neither age nor gender significantly affected ROAM.

**Table 1:** *p*-values, parameter estimates and associated 95 % confidence intervals (CIs): final multiple multilevel regression model

Parameter	Parameter estimate	95 % CI	*p*-value
Constant	27.1	(20.9, 33.2)	<0.001
Age	−0.123	(−0.268, 0.022)	0.096
Gender			
Male (reference)			
Female	2.94	(−0.421, 6.31)	0.086
Footwear			
Barefoot (reference)			
Typical	2.54	(1.46, 3.62)	<0.001
Medical	−1.12	(−2.14, −0.095)	0.032
Garments			
None (reference)			
Bandaging	−2.51	(−3.59, −1.43)	<0.001
Hosiery	0.485	(−0.532, 1.50)	0.350

Hence controlling for age and gender, the use of bandaging was associated with a mean reduction in ROAM of 2.51° at best estimate compared with no garments; the use of typical footwear was associated with a mean increased in ROAM of 2.54° at best estimate compared with barefoot; the use of medical footwear was associated with a mean reduction in ROAM of 1.12° at best estimate compared with barefoot.

## Discussion

The study revealed that compression hosiery outperforms multi-component bandages, which are associated with a significant reduction in ROAM. While there was no evidence for a significant change in ROAM as a result of the application of hosiery, at best estimate a non-significant increase was actually recorded. The effect on ROAM of either type of garment was the same regardless of footwear.

The fact that 14 of the 30 participants could not wear their own typical footwear with the multi-layer compression bandaging reinforces the disadvantage of this treatment when compared to hosiery and highlights the importance of healthcare practitioners coordinating access to specialist footwear and providing the appropriate advice. The provision of medical footwear is limited; only 12 % of patients received the footwear in a community audit [12]. The inability to wear suitable shoes could prevent patients carrying out everyday activities impacting on their social interactions which can be detrimental to their quality of life, especially when the bandaging is applied for an extensive period. While participants in our study were able to wear the multi-component bandages in conjunction with medical footwear, the use of this footwear in itself may restrict any possible dorsiflexion movement at the ankle joint, as the medical shoe has a rigid sole designed to provide stability for patients and improve safety when the foot is swollen or bandaged. Clinicians should recognise that compression hosiery kits do not cause problems with footwear and thus provides an equally effective alternative to bandaging without the associated footwear complications.

Up to 40° plantarflexion and up to 15° dorsiflexion, totalling a range of 55° [22] is the normal expected ROAM at the ankle joint. The ROAM values found in this study varied from 0.19° to 50.8°, dependent on the footwear and garment conditions. When wearing typical footwear, participant ROAM increased when compared to barefoot; possibly due to the fact the study did not take into account the heel height of the participants’ typical shoe which could have influenced the ROAM recorded. In contrast there was a decrease in ROAM found when comparing barefoot with medical footwear.

While neither age nor gender were revealed to be significantly associated with changes in ROAM at the 5 % significance level, substantive effects of both variables were observed, with higher ROAM values reported in younger participants (a reduction of about 0.12° at best estimate is associated with each year of advancing age); and in females, whose mean ROAM at best estimate was about 2.94° greater than in males.

The multilevel methods utilised in this analysis of clustered data avoid potential over-estimates of statistical significance due to anticipated dependency of data items, and also avoid the restrictive assumptions of alternative approaches such as repeated-measures analysis of variance. The multilevel approach also avoids the ecological fallacy through the aggregation of data, and reflects the notions of contextuality (that ROAM varies between participants and may vary differentially with footwear and garment apparel from participant to participant); and of heterogeneity (the modelling of variability between participants, assessments and steps).

Although the analysis was conducted on 30 individuals, the collection of data from multiple assessments per individual, and multiple steps per assessment, led to the collection of over 1500 data points. The emphasis of the analysis was primarily on estimating between-assessment differences (i.e. those differences that can be accounted for by variation in footwear and garments); and only secondarily on between-participant differences (i.e. those differences that can be accounted for by variation in demographic attributes): hence, the study should provide reliable estimates of footwear and garment effects. The existence of substantial components of variance at all three levels of the model structure vindicates the utilisation of the multilevel approach.

### Limitations

A limitation of the current study is that it was conducted on healthy individuals of generally younger age than a typical patient with venous ulceration. It is known that people with venous hypertension have a reduced ROAM; the degree of reduction is related to the severity of venous disease and clinical symptoms [23]. This is as a result of the chronic inflammation associated with venous hypertension which causes not only skin changes but changes in the muscles, nerves and joints [11]. These restrictions on ROAM may be due to a variety of reasons such as oedema, pain, or adaptive gait strategies; but what is not clear is whether these changes are the cause of or the effect of venous disease [23, 24]. The changes of ankle movement in patients with venous disease are thought to potentially increase patient risk of falling [11], but it is also considered that the bulk of compression bandages may additionally impede ankle movement [11, 24].

This study involved the assessment of the gait of participants wearing compression garments over a limited period of time only. Future work in this area could entail an investigation into the long-term effect on gait after garments are removed, which may lead to reduced activation of the calf muscle pump, resulting in increased risk of ulceration.

## Conclusions

This research suggests that compression hosiery kits do not substantively affect ROAM, whereas multi- component compression bandaging have a significantly negative effect on ROAM; which could increase the risk of falling in patients treated for venous leg ulceration.

Hence the use of hosiery kits (JOBST UlcerCARE) has a significant advantage over the use of compression bandages in terms of ankle mobility. The effect on ROAM and subsequent increased risk of falling need to be taken into consideration by practitioners when selecting compression systems for the treatment of venous ulceration.

## Abbreviations

ΔLRS, change in likelihood ratio statistic; 2-D, 2-dimensional; BMI, body mass index; LRS, likelihood ratio statistic; ROAM, range of ankle motion

## Additional file

Additional file 1: Raw data from gait investigation. (XLSX 256 kb)
